# Meconium Aspiration Syndrome, Hypoxic-Ischemic Encephalopathy and Therapeutic Hypothermia—A Recipe for Severe Pulmonary Hypertension?

**DOI:** 10.3390/children11060673

**Published:** 2024-06-01

**Authors:** Deepika Sankaran, Jessa Rose A. Li, Satyan Lakshminrusimha

**Affiliations:** 1 Division of Neonatology, University of California, Davis, Sacramento, CA 95817, USA; dsankaran@ucdavis.edu; 2 Department of Pediatrics, University of California, Davis, Sacramento, CA 95817, USA; jrali@ucdavis.edu

**Keywords:** meconium aspiration syndrome, hypoxic-ischemic encephalopathy, persistent pulmonary hypertension of the newborn, therapeutic hypothermia

## Abstract

Hypoxic-ischemic encephalopathy (HIE) is the leading cause of mortality among term newborns globally. Infants born through meconium-stained amniotic fluid are at risk of developing meconium aspiration syndrome (MAS) and HIE. Simultaneous occurrence of MAS and HIE is a perilous combination for newborns due to the risk of persistent pulmonary hypertension of the newborn (PPHN). Moreover, therapeutic hypothermia (TH), which is the current standard of care for the management of HIE, may increase pulmonary vascular resistance (PVR) and worsen PPHN. Infants with MAS and HIE require close cardiorespiratory and hemodynamic monitoring for PPHN. Therapeutic strategies, including oxygen supplementation, ventilation, use of surfactant, inhaled nitric oxide and other pulmonary vasodilators, and systemic vasopressors, play a critical role in the management of PPHN in MAS, HIE, and TH. While TH reduces death or disability in infants with HIE, infants with MAS and HIE undergoing TH need close hemodynamic monitoring for PPHN.

## 1. Introduction

**Figure 1:**
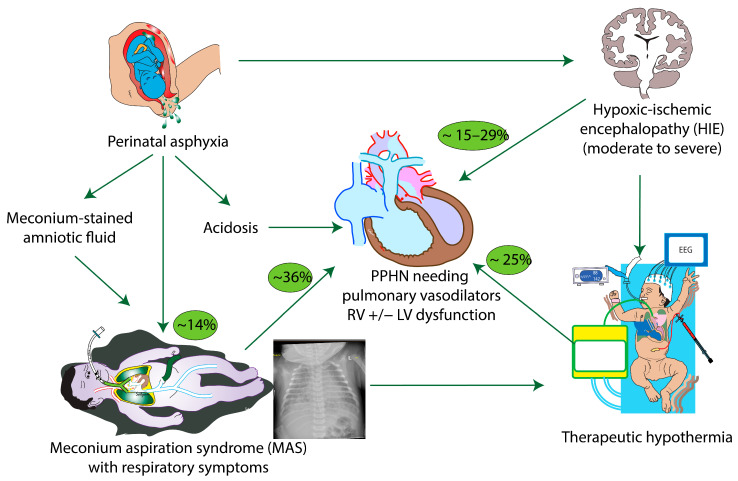
Factors contributing to persistent pulmonary hypertension of the newborn (PPHN) in perinatal asphyxia. The numbers in green ovals indicate the approximate incidence of the condition. For example, 15–29% of infants with moderate to severe HIE have associated PPHN requiring pulmonary vasodilator therapy. Copyright Satyan Lakshminrusimha.

Perinatal asphyxia with hypoxic-ischemic encephalopathy (HIE) is a leading cause of mortality in term infants, accounting for nearly 0.5–1 million deaths worldwide [1,2]. Persistent pulmonary hypertension of the newborn (PPHN), often secondary to meconium aspiration syndrome (MAS), is observed in 20–25% of term infants with moderate to severe HIE [3,4,5] (Figure 1). Infants with moderate to severe HIE are treated with therapeutic hypothermia (TH, 33.5 °C) to improve survival without neurodevelopmental impairment [6,7]. However, hypothermia exacerbates PPHN [7,8], and the combination of MAS, HIE, and TH is increasingly associated with a need for extracorporeal membrane oxygenation (ECMO).

## 2. Meconium Aspiration Syndrome and PPHN

Meconium aspiration syndrome is a respiratory disorder in a newborn infant that is associated with significant morbidity and mortality. Although the delivery room management has changed within the past decade to “no tracheal suctioning” during resuscitation of non-vigorous infants born through meconium-stained amniotic fluid, the incidence of neonatal intensive care admissions for MAS has increased based on observational studies in the United States (U.S) [9,10]. In-utero fetal distress and fetal asphyxia/hypoxia may stimulate and enhance intestinal peristalsis in the fetus, relax the anal sphincter, and allow passage of meconium. In such circumstances, hypoxia induced by asphyxia may induce gasping and aspiration of the meconium-stained amniotic fluid in utero.

Meconium induces localized inflammation in the lungs by an increase in cytokines, interleukins, reactive oxygen species, and lectin and alternate complement pathway activation in the lungs [11]. Moreover, mechanical obstruction of small airways results in ball-valve effect-induced overinflation and risk of air leak syndromes. Alternating areas of hyperinflation and atelectasis with fluffy infiltrates are characteristic of MAS on chest radiographs. Furthermore, meconium inactivates surfactants by changing its chemical properties, thus reducing lung compliance. In addition to compromised ventilation, MAS is often complicated by HIE and PPHN, resulting from impaired fetal-to-neonatal cardiopulmonary transition. Impaired gas exchange due to inadequate ventilation in MAS is followed by hypoxia-induced pulmonary vasoconstriction that sustains the high pulmonary vascular resistance (PVR) as observed in the fetal circulation. Thus, PVR fails to decrease sufficiently in the early postnatal period, and the pulmonary blood flow remains low, further jeopardizing gas exchange in the lungs and contributing to a vicious cycle of worsening PPHN in MAS. Neonates with MAS and PPHN present clinically with respiratory distress, labile hypoxemia (worsening hypoxemia with agitation/stimulation), and differential cyanosis (splitting of oxygen saturation between pre- and post-ductal pulse oximetry). The pre-post ductal difference in pulse oximetry is secondary to right-to-left shunting across the ductus arteriosus, creating a gradient due to higher pre-ductal and lower post-ductal SpO_2_. Persistent elevation of PVR can be complicated by right ventricular dysfunction and, subsequently, left ventricular dysfunction due to interventricular interactions, resulting in worsening hypoxemia and systemic hypotension followed by tissue-level hypoxia and metabolic acidosis (Figure 1).

The combination of chronic in-utero hypoxia and aspiration of meconium into the lungs may often facilitate the development of PPHN in the postnatal period. The simultaneous occurrence of HIE is associated with a precipitous release of inflammatory vasoactive mediators, including TNF-α, interleukin-1β, interleukin-6, and interleukin-10, which are linked to contraction and proliferation of pulmonary artery smooth muscle cells (PASMCs). Meconium by itself can alter cyclooxygenase metabolites and affect nitric oxide synthase (NOS) activity. A decrease in the expression and activity of endothelial NOS was observed in a perinatal lamb model of PPHN. Similarly, reduced expression of NOS was reported in human newborns with PPHN secondary to MAS [12,13,14].

## 3. Hypoxic-Ischemic Encephalopathy and PPHN

While the primary focus following perinatal asphyxia remains on improving the neurodevelopmental outcome after cerebral injury in HIE, the potential harm from inadequate blood flow and hypoxia may extend to the cardiorespiratory system, contributing to morbidity and mortality [15]. Hypoxemic respiratory failure due to PPHN is observed in up to a quarter of the neonates with moderate-to-severe HIE, and about half to two-thirds of these neonates require cardiovascular support for at least 24 h [6,16]. Infants with HIE and PPHN present clinically with respiratory failure with severe hypoxia, V/Q mismatch, low cardiac output, and severe systemic hypotension. The mechanisms underlying the increased risk of PPHN in infants with HIE and its clinical implications are outlined below.

### 3.1. Mechanisms Contributing to PPHN and Cardiac Dysfunction in HIE

#### 3.1.1. Disrupted Fetal to Neonatal Cardiopulmonary Transition

The well-orchestrated fetal-to-neonatal cardiopulmonary transition includes lung inflation with displacement of lung fluid by air, which decreases PVR, increasing pulmonary blood flow by pulmonary vasodilation and reversing the direction of shunting across the ductus arteriosus and foramen ovale (from fetal right-to-left to neonatal left-to-right shunting) is disrupted in infants with perinatal asphyxia and HIE. Therefore, PVR remains persistently elevated, limiting pulmonary blood flow and compromising gas exchange in the lungs. Following the clamping of the umbilical cord, the systemic vascular resistance (SVR) increases sharply with the removal of the low-resistance placental circuit from the systemic circulation [15,17]. The ratio of PVR/SVR will determine the degree of shunting across the ductus arteriosus and foramen ovale.

**Figure 2:**
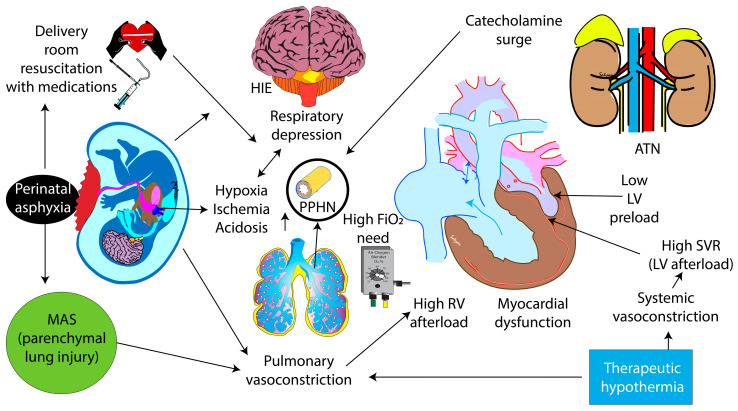
Pathophysiology of PPHN associated with HIE and therapeutic hypothermia. Perinatal asphyxia is associated with hypoxia, ischemia, and acidosis, leading to HIE, PPHN, myocardial dysfunction, and acute tubular necrosis (ATN). The use of epinephrine during resuscitation and an endogenous catecholamine surge can contribute to pulmonary vasoconstriction. Therapeutic hypothermia can exacerbate PPHN by constricting pulmonary vasculature. PPHN—persistent pulmonary hypertension of the newborn; HIE—hypoxic-ischemic encephalopathy; MAS—meconium aspiration syndrome; RV—right ventricle; LV—left ventricle; ATN—acute tubular necrosis; SVR—systemic vascular resistance. Modified from [15]; copyright Satyan Lakshminrusimha—with permission.

During the postnatal period, after a perinatal hypoxic-ischemic insult, there is hypercapnia and metabolic acidosis that can both raise PVR. Hypoxia and acidosis in infants with HIE increase PVR, particularly when associated with prolonged in-utero hypoxia [18]. Hypoxia-induced pulmonary vasoconstriction can contribute to an increase in PVR. This is particularly worse in infants with HIE who also have concurrent respiratory disorders such as MAS that further worsen hypoxia, resulting in a vicious cycle of hypoxia and an increase in PVR (Figure 2) [19]. Furthermore, epinephrine use during resuscitation or a catecholamine surge in infants with HIE may exacerbate PPHN by direct effect of pulmonary vasoconstriction. Due to sustained pulmonary vasoconstriction, ventilation-perfusion mismatch and intra-pulmonary shunting occur, resulting in deoxygenated blood returning to the left ventricle (LV) to be pumped into the systemic circulation.

#### 3.1.2. Myocardial Dysfunction

The immature neonatal myocardium has disordered myofibrils, an underdeveloped calcium-sensing mechanism in their sarcoplasmic reticulum, and less-compliant collagen, contributing to a poorly developed contractile system. Therefore, the fetal/neonatal myocardium functions at the peak of the ventricular function curve with less impact of change in preload and end-diastolic volume on cardiac contractility and cardiac output [20]. When the fetal/neonatal myocardium is subjected to hypoxic-ischemic stress in the setting of an acute perinatal event leading to HIE, the cardiac myocytes are vulnerable to metabolic consequences. The transition from glycolysis-predominant fetal cardiomyocyte metabolism to β-oxidation of fatty acids in the newborn is delayed, thus reducing energy generation in the setting of increased metabolic demand [21].

Newborns with HIE may have depressed LV function from the hypoxic-ischemic insult and from oxidative stress owing to reperfusion injury to the myocardium [22]. Left ventricular dysfunction can present clinically with evidence of heart failure, reduced cardiac output, and pulmonary venous hypertension (elevated pulmonary capillary wedge pressure). High PVR and reduced pulmonary blood flow are accompanied by reduced pulmonary venous return to the left atrium, thus reducing LV preload and cardiac output. Additionally, depressed right ventricular function due to direct hypoxic-ischemic insult to the right ventricle (RV) can occur following HIE. In the setting of HIE and PPHN, the failing RV is unable to pump deoxygenated blood into the pulmonary artery against a high PVR and PAP (or high RV afterload), further compromising pulmonary blood flow and oxygenation. In order to sustain pulmonary blood flow in the setting of RV dysfunction, the RV adapts by muscular hypertrophy to enhance its ability to contract. Prolonged exposure to high afterload may push the RV towards maladaptation and dilation with a decrease in forward blood flow into the pulmonary artery. This is followed by interventricular septal bowing, underfilling, dysfunction of the compressed LV, and an increase in heart rate in an attempt to improve pulmonary and systemic blood flow [23]. Complex interventricular interactions ensue, further decreasing LV filling and cardiac output. Finally, global ventricular dysfunction may be followed by biventricular enlargement, dilated cardiomyopathy, poor systemic blood flow, and end-organ ischemia.

## 4. MAS, HIE and TH

Infants with MAS often have HIE requiring TH as the standard of care to reduce the risk of death or disability [8]. MAS interferes with gas exchange, impairs lung compliance, worsens hypoxia, and increases PVR. When there is concurrent HIE with MAS, there is an exacerbation of PPHN due to hypoxic vasoconstriction, hypercarbia and acidosis, and myocardial dysfunction, as described above. Additionally, TH, which is the current standard of care for the management of moderate to severe HIE in newborns, can worsen PPHN. The cardiovascular effects of the combination of MAS, HIE, and TH are outlined below.

### 4.1. MAS and HIE—A Precarious Combination

The passage of meconium in utero is stimulated by fetal stress or asphyxia [24]. Chronic in-utero hypoxia induces gasping in the fetus that allows meconium to be aspirated into the lungs even prior to birth. Exposure to a combination of aspirated meconium and hypoxia may stimulate pulmonary arterial smooth muscle hypertrophy even prior to birth. This exposure to meconium in-utero can lead to injury to lung parenchyma and affect the molecular mechanisms regulating pulmonary vascular tone. Additionally, respiratory depression that is characteristic of MAS worsens hypoxemia, further contributing to high PVR in these infants with HIE. While MAS and HIE can separately be complicated by PPHN, their concurrent occurrence can worsen the severity of PPHN, making this a clinical challenge. While gentle ventilation strategies and the use of oxygen supplementation are critical in the management of MAS + PPHN, hypocapnia, and hyperoxia may worsen outcomes in HIE [25]. On the contrary, therapeutic hypothermia, which is the current standard of care in the management of moderate-to-severe HIE, can worsen PPHN by increasing PVR in infants with MAS. Thus, factors that are potentially neuroprotective, such as therapeutic hypothermia, permissive hypercapnia, and limiting oxygen, may potentially exacerbate PPHN. In contrast, normothermia, oxygen, and avoiding hypercarbia promote pulmonary vasodilation.

### 4.2. Cardiovascular Effects of TH

Therapeutic hypothermia to 33.5 °C initiated within 6 h after birth and continued for 72 h is the standard of care in the management of infants ≥ 36 weeks with moderate to severe HIE in high-income countries (HIC). This is owing to the neuroprotective effects of TH on pathways that contribute to secondary brain injury in HIE, including excitatory amino acids, cerebral blood flow and metabolism, cerebral energy state, nitric oxide production, and apoptosis [8]. TH is a proven intervention that reduces the risk of death or disability in infants with moderate or severe HIE. However, TH in MAS + HIE may be associated with adverse cardiopulmonary effects that may impact survival and long-term neurodevelopmental outcomes, and these associations have not been evaluated extensively [26]. Hypothermia increases the risk of PPHN, a condition common among patients with asphyxia [24], and 25% of infants in the whole-body hypothermia trials had evidence of PPHN [7,8,27]. Hypothermia induces pulmonary vasoconstriction and increases PVR in animal models (2819 ± 239 dyn.s.cm^−5^ at 37 °C to 3696 ± 441 dyn.s.cm^−5^ at 34.6 °C and 4201 ± 517 dyn.s.cm^−5^ at 31.2 °C) [28]. The increase in PVR during hypothermia may be attributed to direct effects of pulmonary vasoconstriction and potentially related to increased blood viscosity and catecholamine responses [15].

Randomized clinical trials have not shown an increase in PPHN with TH (RR 1.36, 0.95 to 1.96) [29]. However, subjects in the Eunice Kennedy Shriver National Institute of Child Health and Human Development (NICHD) trials had high PaO_2_ (132 ± 145 mm Hg) and FiO_2_ (0.84 ± 0.16) during the intervention period that may have reduced the incidence of PPHN [6]. Contrary to randomized trials, hypothermia outside clinical studies is associated with a higher incidence of PPHN [30]. Approximately 23–25% of infants cooled for moderate to severe HIE have PPHN, and 39% of infants with MAS cooled for HIE have PPHN [6,8]. A recent clinical observational study that included ten neonates treated with TH for HIE reported increased PAP during TH [31]. Randomized clinical trials are warranted to assess the clinical and hemodynamic impact of TH on PPHN in the setting of MAS and HIE.

Although TH is the standard of care for moderate to severe HIE in HIC in term infants, uncertainty persists about the efficacy of TH in low-resource settings and low-and-middle-income countries (LMIC). In the HELIX trial evaluating TH in LMIC, Thayyil et al. reported increased deaths during neonatal hospitalizations in the TH group (36% vs. 24%, *p* = 0.0087) [32]. Although PPHN was slightly more common with TH, this increase was not statistically significant in this study [32]. The high incidence of MAS, pneumonia, and sepsis associated with perinatal asphyxia and the lack of resources to treat PPHN in LMIC may be one of the factors accounting for the difference in TH outcomes between HIC and LMIC. However, two other studies from India evaluating TH did not demonstrate an increase in PPHN with TH [33,34].

In addition to the increase in PVR, TH may be associated with impaired LV and RV contractility, reduced LV and RV preload, lowered heart rate, and increased SVR [15]. Right ventricular function may be reduced during TH both due to ischemic injury to the cardiomyocyte and reduced metabolic demand during TH. This can lead to RV failure when increasing RV afterload in PPHN. Depressed LV function in HIE may be complicated by systemic hypotension. An increase in SVR induced by TH due to the direct effects of peripheral vasoconstriction counteracts the increased vascular permeability in HIE. Sinus bradycardia is often observed during TH, which is likely due to slow diastolic repolarization in the sinoatrial node, prolonged conduction time, and suppressed sympathetic stimulation [35]. Although sinus bradycardia can be cardioprotective by reducing the metabolic demand of cardiomyocytes, it can be accompanied by a significant reduction in cardiac output without a significant change in superior vena caval flow, indicating preserved cerebral perfusion [36]. This preserved or high superior vena caval flow is probably secondary to vasoparalysis [37] and may be associated with MRI changes [36].

### 4.3. Cardiorespiratory Effects of TH

Poor oxygenation characteristics of MAS, HIE, and PPHN may be inadvertently managed with excessive mean airway pressures on the ventilator. Use of high mean airway pressures leads to overdistension of respiratory bronchioles and alveoli, thereby impeding the pulmonary blood flow and gas exchange. Additionally, low temperature shifts the oxygen–hemoglobin dissociation curve to the left and confounds pulse oximetry-based assessment of arterial partial pressure of oxygen (PaO_2_). Data from 56 neonates with HIE who had 385 arterial blood gases drawn at 37 and 33.5 °C with simultaneous SpO_2_ recordings suggests that pulse oximetry often overestimates PaO_2_ during hypothermia. In addition, adjusting blood gases to the patient’s actual body temperature during hypothermia decreases corrected PaCO_2_ levels. It is important to use temperature-corrected arterial blood gases to avoid hypocapnia, reduced cerebral blood flow, and uneven cooling of the brain, which may potentially limit neuroprotection. All clinical trials of whole-body cooling utilized temperature-corrected blood gases for adjusting ventilation.

## 5. Optimal Therapeutic Strategies for PPHN in the Setting of MAS, HIE and TH

### 5.1. Oxygen Saturation Targets

**Figure 3:**
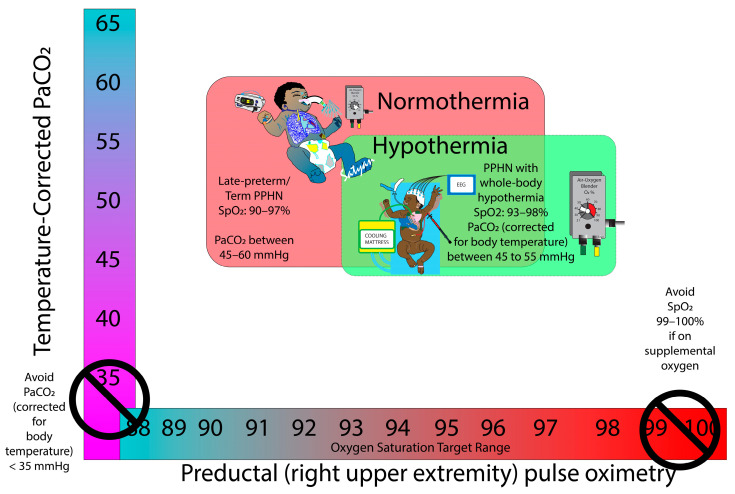
Oxygen saturation and PaCO_2_ parameters during the management of PPHN in normothermia and hypothermia. Modified from Lakshminrusimha and Abman, Clin in Perinatology 2024 [41]. Copyright Satyan Lakshminrusimha—with permission.

Oxygen is a pulmonary vasodilator and cerebral vasoconstrictor [38]. While hypoxemia can worsen PPHN by hypoxic pulmonary vasoconstriction, hyperoxia may be associated with oxygen-free radical-mediated oxidative injury to cerebral tissue in addition to the preexisting hypoxic-ischemic and reperfusion injury. Mechanical ventilation with supplemental oxygen and targeting preductal SpO_2_ of 91–95% is currently recommended by guidelines from the European Pediatric Pulmonary Vascular Disease Network for the management of HRF/PPHN. The American Heart Association (AHA) and American Thoracic Society (ATS) suggest initiating oxygen therapy at SpO_2_ < 92% for pulmonary arterial hypertension [39] and at SpO_2_ of 92–94% for infants with bronchopulmonary dysplasia with pulmonary hypertension. These recommendations are based on expert opinion and not specific to PPHN associated with hypothermia. We speculate that targeting SpO_2_ in the low 90s during TH may exacerbate PPHN due to persistently elevated PVR secondary to lower alveolar and arterial PO_2_. On the contrary, increasing inspired supplemental oxygen will increase the alveolar oxygen tension that will promote pulmonary vasodilation and decrease PVR. We suggest targeting 93–98% preductal SpO_2_ to avoid hypoxemia and hyperoxemia while optimizing pulmonary blood flow and avoiding cerebral and pulmonary oxidative stress (Figure 3). High SpO_2_ of 99–100% must be avoided while on supplemental oxygen, as hyperoxia (PaO_2_ > 100–115 mmHg) in the first few hours of the postnatal period is associated with a higher incidence of HIE following perinatal distress [40].

### 5.2. Ventilation Strategies, Surfactant and iNO

Management of PPHN in MAS and HIE entails selective pulmonary vasodilation, which is usually achieved by lung recruitment, surfactant administration, and inhaled nitric oxide therapy. Lung recruitment maneuvers include increasing positive end expiratory pressure (PEEP) in conventional ventilation or mean airway pressure in high-frequency ventilation. Due to heterogenous lung disease with alternating areas of atelectasis and hyperinflation that are characteristic of MAS, these infants are predisposed to air leak syndromes (such as pneumothorax, pneumomediastinum, pulmonary interstitial emphysema, pneumopericardium, etc.), the risk of which increases further with raising the mean airway pressure. The use of high-frequency ventilation (such as a jet or oscillator) may allow us to achieve higher mean airway pressures without causing significant barotrauma and volutrauma and subsequent complications of air leak syndromes.

#### 5.2.1. Surfactant and Inhaled Nitric Oxide

Meconium can inactivate surfactant, worsening atelectasis in affected areas of the lung. There is a role for surfactant administration and/or surfactant lavage in the management of neonates with MAS. Surfactant administration will replenish surfactant, reduce surface tension in affected alveoli, and improve gas exchange. In some cases, surfactant administration enables the suctioning of meconium plugs, enhancing ventilation. Administration of surfactant enhances the effect of inhaled nitric oxide (iNO) [42]. However, interventions to improve lung aeration without addressing the high PVR limiting pulmonary blood flow in infants with MAS, HIE, and PPHN may result in V/Q mismatch due to aerated areas of the lung that are not perfused (not contributing to gas exchange). Inhaled nitric oxide at a dose of 20 ppm is a selective pulmonary vasodilator that acts via the cGMP pathway to relax the PASMCs [43]. iNO can be administered to infants both on invasive and non-invasive ventilation and usually does not cause adverse effects on SVR. On entering the pulmonary circulation, NO is inactivated by hemoglobin to form methemoglobin, thus minimizing systemic vasodilation and limiting extrapulmonary right-to-left shunting [44]. Additionally, iNO induces vasodilation in ventilated airways and improves V/Q matching. In an elegant post-hoc analysis of the iNO-randomized controlled trial by Konduri et al., early use of surfactant and iNO in infants with moderate hypoxic respiratory failure (oxygenation index 15–25) is associated with decreased risk of ECMO/death, progression of HRF and decreased length of hospital stay [45]. iNO should be avoided in infants with LV dysfunction as it may precipitate pulmonary edema.

#### 5.2.2. Targets for pH and PaCO_2_

Hypercarbia, hypoxia, and acidosis that may raise PVR should be avoided in infants with MAS, HIE, and PPHN [46]. In infants with PPHN not undergoing hypothermia, ventilation strategies should include targeting normoxemia (SpO_2_ 90–97%, PaO_2_ 50–80 mmHg), permissive hypercapnia (PaCO_2_ in the 45–60 range) and pH > 7.25, preferably 7.30–7.40 [4]. Hyperventilation and hypocapnia should be avoided because of the risk of reduced cerebral perfusion and sensorineural hearing loss in the setting of HIE and PPHN [4]. In the presence of hypothermia, although the PaO_2_ target remains the same (50–80 mmHg), the preductal SpO_2_ target needs to be higher at 93–98% due to the shift in the oxygen–hemoglobin dissociation curve to the left. In addition, as acidosis and hypothermia exacerbate pulmonary vasoconstriction, we recommend maintaining temperature-corrected PaCO_2_ levels within a tight range of 45–55 mmHg in patients with PPHN on whole-body hypothermia.

#### 5.2.3. Sodium Bicarbonate

**Figure 4:**
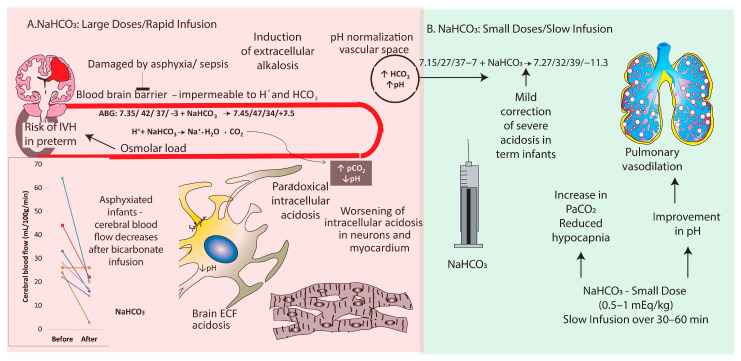
Harmful effects of large doses of sodium bicarbonate infused rapidly in asphyxiated infants with PPHN ((**A**) red section on the left side of the figure). Although plasma bicarbonate and pH increase, alkalosis can lead to intracellular acidosis, especially in neurons and cardiac myocytes. Each of the colored lines in the inset graph represent individual patient on change in cerebral blood flow before and after rapid sodium bicarbonate bolus. However, in the presence of severe acidosis and hypocapnia associated with HIE, a slow infusion of a small dose of sodium bicarbonate may help reduce hypocapnia, mildly correct acidosis, and lead to pulmonary vasodilation in HIE with PPHN ((**B**) green section on the right side). Copyright Satyan Lakshminrusimha.

Sodium bicarbonate is typically avoided in the management of metabolic acidosis in the NICU due to its potential to aggravate intramyocardial acidosis [47,48]. Large doses with rapid infusion can also lead to osmolar load and increase the risk of intraventricular hemorrhage in preterm infants. However, in rare instances, when ventilated term infants with HIE have metabolic acidosis and hypocapnia secondary to Kussmaul breathing. Hypocapnia decreases cerebral blood flow and is associated with an increased risk of death or neurodevelopmental impairment in HIE. A small dose of sodium bicarbonate (0.5 to 1 mEq/kg) administered slowly over 30 min may potentially increase PaCO_2_ and pH and decrease PVR. However, there are no randomized trials evaluating bicarbonate therapy in HIE or PPHN. Higher doses of sodium bicarbonate, especially when infused at rapid rates (0.5 mEq/min), are associated with a reduction in cerebral blood flow in asphyxiated term neonates and should be avoided [49]. Induction of alkalosis with large doses of sodium bicarbonate, as was the practice in the management of PPHN in the 1980s, can lead to cerebral vasoconstriction [50] and poor neurological outcomes [51,52]. The formation of CO_2_ following the interaction of sodium bicarbonate with H+ ions can add to respiratory acidosis in patients with inadequate ventilation. CO_2_ can also cross the blood–brain barrier causing brain extracellular fluid (ECF) acidosis, and cross the cell membrane to cause paradoxical intracellular acidosis (Figure 4). However, there may be potential benefits of small doses of sodium bicarbonate infused slowly to correct base deficit and increase pH > 7.25 in PPHN and some cases of congenital heart disease (Figure 4) [53]. Cases of HIE with hyperventilation due to cerebral irritation and low PaCO_2_ secondary to metabolic acidosis can potentially benefit from a small dose (0.5 to 1 mEq/kg) of sodium bicarbonate infused slowly over 30 min to an hour.

### 5.3. Systemically Administered Pulmonary Vasodilators

Infants with MAS, HIE, and PPHN who are minimally responsive to optimized ventilation, supplemental oxygen, and iNO may require additional agents to reduce PVR (pulmonary vasodilators such as sildenafil, prostaglandins, bosentan) and/or improve RV function (inodilators, such as milrinone).

#### 5.3.1. Sildenafil

**Figure 5:**
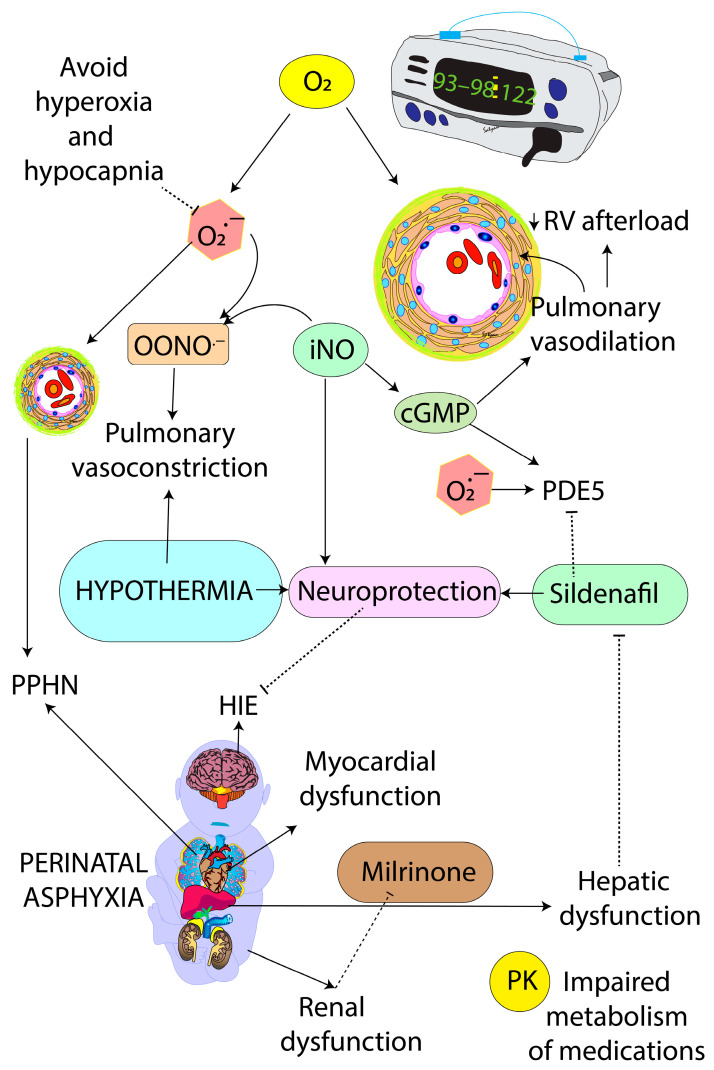
Pulmonary vasodilators in HIE with PPHN during therapeutic hypothermia. Sildenafil may have a dual role in enhancing pulmonary vasodilation while being neuroprotective. Milrinone similarly may have a dual role in pulmonary vasodilation and enhancing cardiac function. However, the pharmacokinetics (PK) of these medications should be closely monitored as hepatic dysfunction and renal dysfunction can increase the levels of sildenafil and milrinone, respectively, increasing the risk of hypotension. OONO^•−^ (peroxynitrite—a toxic compound resulting from the combination of nitric oxide and superoxide anions (O_2_^•−^) that can contribute to oxidative and nitrosative stress in HIE and PPHN. Copyright Satyan Lakshminrusimha.

Sildenafil is a phosphodiesterase-5 inhibitor that increases the cGMP concentrations in the PASMCs, resulting in pulmonary vasodilation. When administered intravenously (loading dose (0.14 mg/kg/h over 3 h, followed by 0.07 mg/kg/h continuous infusion), sildenafil also has effects on systemic vascular bed manifesting as severe systemic hypotension. Sildenafil may also have neuroprotective effects in infants with HIE [54]. Pilot trials are currently underway to evaluate sildenafil in HIE [55]. If found to be effective as a neuroprotective agent in HIE, oral sildenafil has the potential to improve pulmonary and neurological outcomes in HIE with PPHN in resource-limited settings (Figure 5). Due to hepatic dysfunction in many infants with HIE, lower doses of sildenafil may be optimal, and we recommend half the above dose (0.07 mg/kg load over 3 h followed by 0.035 mg/kg/h) and then titrate the dose based on systemic blood pressure and oxygenation response.

#### 5.3.2. Milrinone

Milrinone is a phosphodiesterase-3 inhibitor that acts by increasing cAMP concentrations in the PASMCs, resulting in pulmonary vasodilation. Due to its non-selective effects on the systemic and pulmonary vascular beds, systemic hypotension is a known complication of milrinone. Milrinone (0.2–1 mcg/kg/min) additionally has a positive inotropic effect on the left and right ventricles by improving myocyte contractility, better lusitropy, and improves LV and RV performance by afterload reduction for both the LV and RV [43]. Furthermore, the use of milrinone can reduce right-to-left shunting across the ductus arteriosus and foramen ovale, benefit oxygenation, and enhance the response to iNO. Milrinone is cleared by the kidneys and may accumulate in the circulation in infants with MAS and HIE, as well as acute tubular necrosis (ATN) secondary to HIE. In cases of renal dysfunction, we recommend lower doses of milrinone (0.2 to 0.33 mcg/kg/min) without a bolus and titrate based on systemic blood pressure. Higher doses of milrinone in HIE and ATN can lead to systemic hypotension during therapeutic hypothermia [56].

#### 5.3.3. Prostaglandin I_2_ (PGI_2_)

Inhaled (epoprostenol) and intravenous (epoprostenol and treprostinil) PGI_2_ act to reduce PVR by activation of adenylate cyclase and increasing intracellular cAMP concentrations in PASMCs [57,58]. The addition of PGI_2_ may improve oxygenation in infants with PPHN that are refractory to iNO and other pulmonary vasodilators.

#### 5.3.4. Bosentan

Bosentan is an endothelin (ET-1) receptor antagonist that is administered enterally in the management of infants with iNO refractory PPHN. The acute use of bosentan warrants more studies due to the lack of benefit in a randomized controlled trial of the early addition of bosentan to iNO [59].

#### 5.3.5. Riociguat

Riociguat is an enteral soluble guanylyl cyclase activator that is currently approved to manage chronic pulmonary hypertension in adults [60]. It is being considered for use as a novel therapy in PPHN and warrants clinical trials [61].

### 5.4. Management of Systemic Hypotension in PPHN in MAS and HIE during TH (Figure 6)

Low SVR and systemic hypotension are commonly observed in infants with PPHN and lead to end-organ injury from poor perfusion and hypoxemia [62,63]. Two-thirds of infants with PPHN requiring ventilation and 87% of those requiring extracorporeal life support (ECLS) have systemic hypotension requiring three or more vasopressor medications [64]. Most centers start with a crystalloid bolus (typically normal saline) to treat systemic hypotension in PPHN, attributing the low systemic BP to low intravascular volume status. There is significant variation in the choice of subsequent vasopressor use.

Overemphasis on raising the systemic arterial pressure (SAP) may distract the clinician from the primary physiological disturbance, i.e., elevated PVR. Increasing SAP may increase the strain on the RV and decrease cardiac output and SAP (owing to interventricular interactions between the RV and LV) [62]. Low SAP is the primary driver of vasopressor selection [65,66]. Dopamine is often the first choice to manage neonatal systemic hypotension, irrespective of the underlying physiology, due to its familiarity, simple first-order pharmacokinetics, ability to quickly achieve steady-state levels, and presumed safety profile [65]. However, in addition to the desired effect of dopamine receptor-mediated systemic vasoconstriction, dopamine also causes β1-mediated positive inotropic, chronotropic, and dromotropic effects on the heart. In contrast, norepinephrine causes α1-mediated systemic vasoconstriction and possible α2-mediated pulmonary vasodilation. Similarly, vasopressin is a potent systemic vasoconstrictor acting via the V1 receptors in systemic circulation. Vasopressin may have pulmonary vasodilator effects through V1 receptors in the pulmonary vascular bed by releasing endothelial NO. However, vasopressin use in infants with HIE can be associated with undesired effects, including hyponatremia, fluid retention, and oliguria secondary to ATN in HIE. Epinephrine raises SVR by α1-mediated systemic vasoconstriction and has β1-mediated positive inotropic, chronotropic, dromotropic, and lusitropic effects on the heart.

**Figure 6:**
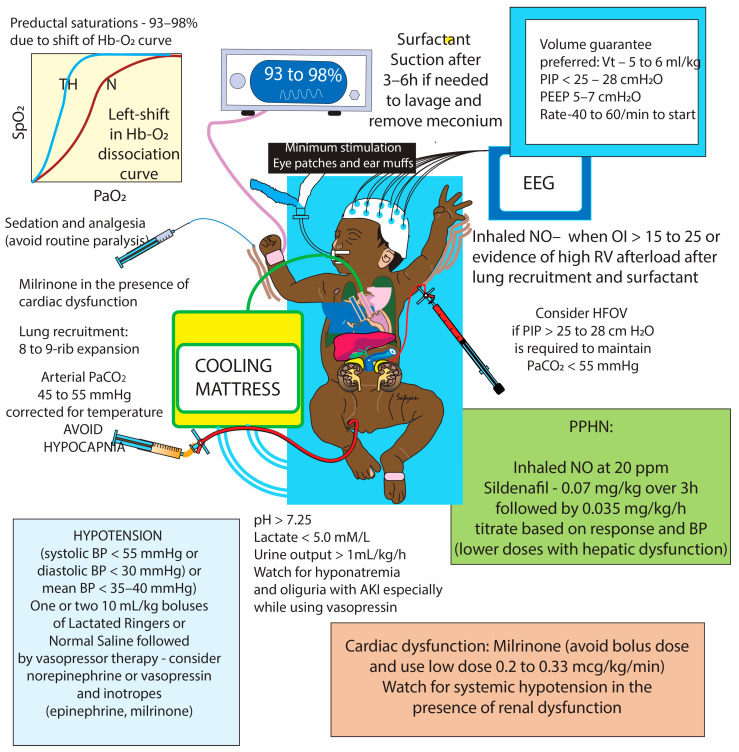
Management of PPHN and HIE with MAS during therapeutic hypothermia. Selective pulmonary vasodilation is achieved by lung recruitment (as depicted above), surfactant administration (to overcome inactivation by surfactant), and inhaled nitric oxide. Targeting preductal SpO_2_ of 93–98% (due to the left-shift of the hemoglobin–O_2_ dissociation curve during hypothermia) may optimize pulmonary blood flow and avoid oxidative stress. Tight control of PaCO_2_ at 45–55 mm Hg during therapeutic hypothermia may prevent a reduction in cerebral blood flow secondary to hypocapnia and an increase in pulmonary vascular resistance secondary to hypercapnia and respiratory acidosis. Sildenafil and milrinone are pulmonary vasodilators that have systemic vasodilatory effects causing systemic hypotension. Milrinone is used to manage cardiac dysfunction in infants in MAS, HIE, and PPHN. Lower doses of sildenafil and milrinone are suggested in infants with hepatic and renal dysfunctions (secondary to acute kidney injury/AKI in infants with HIE), respectively. Minimal stimulation, eye patches, and optimized sedation can help to avoid an increase in PVR due to reactive pulmonary vasculature. Routine paralysis should be avoided to prevent systemic hypotension as a complication. Management of systemic hypotension includes 1–2 fluid boluses followed by initiation of norepinephrine or vasopressin for diastolic hypotension (by an increase in systemic vascular resistance) or use of inotropes such as epinephrine for systemic hypotension. The use of vasopressin may be complicated by fluid retention and hyponatremia, especially in the setting of AKI in HIE. Copyright Satyan Lakshminrusimha.

Strategies to achieve supranormal SAP in an attempt to reverse the right-to-left shunt across the foramen ovale and ductus arteriosus to improve oxygenation may prove to be deleterious by inadvertently raising PVR and worsening PPHN [22]. Initiating and titrating vasopressors targeting higher SAP can increase the afterload on the LV and also on the RV myocardium, which is quite sensitive to the afterload [67]. While Feltes et al. reported an increase in SVR without altering PVR with the use of 0–160 µg/kg/min of dopamine in 5–12 day-old lambs, Cheung et al. reported an increase in both SAP and PAP and SAP/PAP ratio with use of 0–32 µg/kg/min of dopamine in 1–3-day old piglets [68,69]. We previously described the selective increase in SAP with dopamine in control lambs but a simultaneous increase in SAP and PAP without altering the SAP/PAP ratio with the use of dopamine in lambs with PPHN, indicating the harmful effect on PVR after dopamine infusion [17]. Similarly, Manouchehri et al. reported a 101% increase in the PAP/SAP ratio from baseline with the use of dopamine (20 µg/kg/min) in asphyxiated piglets [70]. In this circumstance, a systemic vasoconstrictor that is sparing to the pulmonary vascular bed may be an ideal agent to restore SAP and cardiac output without increasing PVR (thus increasing the SVR/PVR ratio). Limited neonatal evidence supports norepinephrine use, although its physiological effects may prove beneficial in PPHN. Tourneux et al. demonstrated an increase in SAP and LV output and marginal improvement in oxygenation following norepinephrine use in an observational study [71]. In contrast, in porcine hemorrhagic shock, norepinephrine reduced the SVR/PVR ratio, while vasopressin increased the ratio [72]. Epinephrine is less selective than norepinephrine; its effect on α and ẞ receptors varies by dose and may be beneficial in myocardial dysfunction at lower doses. However, epinephrine has been shown to increase PAP incrementally in animal models [73]. Mohamed et al. showed improved oxygenation with the use of vasopressin in term infants with hypoxemic respiratory failure and refractory PPHN [74]. In animal studies, low-dose vasopressin causes selective vasodilatation in pulmonary, renal, coronary, and cerebral vasculature under hypoxic conditions while causing vasoconstriction in other vascular beds [75,76,77]. Furthermore, vasopressin decreases PVR by release of endothelial NO in normal or hypoxic conditions [78,79,80].

Commonly used vasopressors in newborns, such as dopamine, norepinephrine, epinephrine, and vasopressin, have variable effects on both pulmonary and systemic vascular beds that are not clearly understood in PPHN [81]. While dopamine and epinephrine can increase SVR by systemic vasoconstriction, they may increase PVR by directly vasoconstrictor effects on the pulmonary vascular bed, thus worsening PPHN [65]. On the contrary, some clinical studies have suggested that norepinephrine and vasopressin cause systemic vasoconstriction but may potentially induce pulmonary vasodilation and may be beneficial in PPHN [81]. The ideal vasopressor that is relatively selective to the systemic circulation and increases the SVR/PVR ratio in PPHN is not known. While obtaining additional physiological information by means of targeted neonatal echocardiography may provide critical information that may guide clinical management in infants with MAS, HIE, and PPHN, its superiority to clinical assessments alone needs to be proved by prospective randomized studies in various clinical conditions in critically ill neonates [22]. For the management of systemic hypotension without cardiac dysfunction, norepinephrine is our suggested first-choice medication in the presence of HIE, PPHN, and AKI during hypothermia. If there is co-existing ventricular dysfunction with systemic hypotension, we prefer epinephrine.

Hydrocortisone is often used in the management of both refractory pulmonary hypertension and severe systemic hypotension in PPHN despite the lack of randomized trials supporting its use. Use of hydrocortisone improved oxygenation index in infants with PPHN in a retrospective study by Alsaleem et al. [82]. In the absence of sepsis, considering a replacement dose of hydrocortisone might enhance catecholamine action and avoid catecholamine-resistant hypotension.

### 5.5. Extracorporeal Membrane Oxygenation

Newborns with MAS and HIE with refractory PPHN unresponsive to medical interventions may be considered candidates for ECMO. ECMO has improved survival for infants with reversible causes of PPHN, such as MAS and HIE [83]. There is considerable debate in babies undergoing hypothermia and having severe PPHN at the brink of ECMO. In these patients, some centers initiate rewarming to improve pulmonary vasodilation. We prefer cannulation for ECMO with the circuit cooled to 33.5 degrees C to continue hypothermia. Meticulous attention must be paid to managing anticoagulation during ECMO with a hypothermic circuit.

## 6. Long-Term Outcomes

Infants with MAS and HIE with PPHN are at risk of neurodevelopmental impairment, including delay in cognitive and motor performance, cerebral palsy, and sensorineural hearing loss [84]. Term infants with PPHN (excluding CDH) are at risk of neurodevelopmental impairment in up to 25% of the infants at 18–24 months of age, 9.2% in school age, and sensorineural hearing loss in about 6.4% of the children at school age [84,85].

Infants requiring management with ECMO are at risk of neurodevelopmental impairment, but the outcomes are more influenced by the underlying etiology rather than ECMO itself [84]. Additionally, those infants with MAS and HIE may require follow-up with pediatric pulmonary hypertension specialists due to the need for outpatient pulmonary vasodilator therapy and pediatric pulmonology if there is a need for home oxygen therapy. Infants with MAS, HIE, and PPHN are at risk of mortality, which can be averted by the use of newer pulmonary vasodilator therapies and ECMO.

## 7. Conclusions

Infants with MAS and HIE are a specialized cohort who are at risk of developing PPHN. These infants require close cardiovascular monitoring. Hemodynamic assessments with the help of targeted neonatal echocardiograms may help in the early recognition of PPHN phenotypes and guide in choosing appropriate therapeutic strategies that improve pulmonary and neurological outcomes. Avoidance of hypoxemia, hypercarbia, and acidosis may potentially minimize the severity and worsening of PPHN in MAS and HIE treated with TH. Early recognition of PPHN and therapy with appropriate pulmonary vasodilators such as iNO, sildenafil, and milrinone may potentially reduce the adverse effects associated with PPHN. Furthermore, ensuring tight maintenance of temperature control while avoiding severe hypothermia and hyperthermia is critical. Additionally, TH should not be initiated in infants with MAS and mild HIE, outside of randomized trials, who do not currently qualify for TH. Future studies are warranted to assess the impact of TH on long-term cardiovascular outcomes in infants with MAS and HIE. Novel scoring tools to gauge the severity of hypoxemic respiratory failure in PPHN incorporating clinical, laboratory, and echocardiographic parameters will need to be investigated [86].

## Data Availability

The original contributions presented in the study are included in the article, further inquiries can be directed to the corresponding author.
